# Imaging heterogeneity of peptide delivery and binding in solid tumors using SPECT imaging and MRI

**DOI:** 10.1186/s13550-016-0160-4

**Published:** 2016-01-14

**Authors:** J. C. Haeck, K. Bol, C. M. A. de Ridder, L. Brunel, J. A. Fehrentz, J. Martinez, W. M. van Weerden, M. R. Bernsen, M. de Jong, J. F. Veenland

**Affiliations:** Department of Radiology, Erasmus MC, Rotterdam, the Netherlands; Department of Medical Informatics, Erasmus MC, Rotterdam, the Netherlands; Department of Nuclear Medicine, Erasmus MC, Dr. Molewaterplein 50, 3015 GE Rotterdam, the Netherlands; Department of Urology, Erasmus MC, Rotterdam, the Netherlands; IBMM, UMR 5247, CNRS, ENSCM, Faculté de Pharmacie, Université Montpellier, Montpellier, France

**Keywords:** DCE-MRI, SPECT, Tumor targeting, Peptide delivery, Prostate cancer

## Abstract

**Background:**

As model system, a solid-tumor patient-derived xenograft (PDX) model characterized by high peptide receptor expression and histological tissue homogeneity was used to study radiopeptide targeting. In this solid-tumor model, high tumor uptake of targeting peptides was expected. However, in vivo SPECT images showed substantial heterogeneous radioactivity accumulation despite homogenous receptor distribution in the tumor xenografts as assessed by in vitro autoradiography. We hypothesized that delivery of peptide to the tumor cells is dictated by adequate local tumor perfusion. To study this relationship, sequential SPECT/CT and MRI were performed to assess the role of vascular functionality in radiopeptide accumulation.

**Methods:**

High-resolution SPECT and dynamic contrast-enhanced (DCE)-MRI were acquired in six mice bearing PC295 PDX tumors expressing the gastrin-releasing peptide (GRP) receptor. Two hours prior to SPECT imaging, animals received 25 MBq ^111^In(DOTA-(βAla)2-JMV594) (25 pmol). Images were acquired using multipinhole SPECT/CT. Directly after SPECT imaging, MR images were acquired on a 7.0-T dedicated animal scanner. DCE-MR images were quantified using semi-quantitative and quantitative models. The DCE-MR and SPECT images were spatially aligned to compute the correlations between radioactivity and DCE-MRI-derived parameters over the tumor.

**Results:**

Whereas histology, in vitro autoradiography, and multiple-weighted MRI scans all showed homogenous tissue characteristics, both SPECT and DCE-MRI showed heterogeneous distribution patterns throughout the tumor. The average Spearman’s correlation coefficient between SPECT and DCE-MRI ranged from 0.57 to 0.63 for the “exchange-related” DCE-MRI perfusion parameters.

**Conclusions:**

A positive correlation was shown between exchange-related DCE-MRI perfusion parameters and the amount of radioactivity accumulated as measured by SPECT, demonstrating that vascular function was an important aspect of radiopeptide distribution in solid tumors. The combined use of SPECT and MRI added crucial information on the perfusion efficiency versus radiopeptide uptake in solid tumors and showed that functional tumor characteristics varied locally even when the tissue appeared homogenous on current standard assessment techniques.

**Electronic supplementary material:**

The online version of this article (doi:10.1186/s13550-016-0160-4) contains supplementary material, which is available to authorized users.

## Background

The discovery of cell-surface receptors overexpressed in tumors has led to the development of tumor-targeting radiopeptides for a variety of cancers. These radiopeptides are being used for both diagnostic imaging and therapy-response monitoring as well as for tumor-targeted therapy. In order to be sensitive and effective, targeting should be highly specific and distribution of the target should ideally be homogenously distributed among the tumor cells in both the primary and metastatic lesions.

However, heterogeneity of cancer is often present and a well-known negative prognostic factor for therapy outcome [1, 2]. Heterogeneous tumors tend to be more resistant to therapy and more likely to have an aggressive phenotype. Next to the routine study of morphologic tissue appearance, it is also crucial to study functional tissue properties, as heterogeneity in functional characteristics, e.g., vasculature, are also prognostic markers. Current clinical studies that use tumor-targeting radiolabeled peptides are unable to explain potential variable responses to be caused by the degree of homogenous or heterogeneous intra-tumoral distribution of radiopeptides in patients as nuclear imaging techniques do not have sufficient spatial resolution [3, 4]. The influence of intra-tumoral peptide heterogeneity on therapeutic efficacy has as yet not been studied due to these spatial restrictions and remains an important issue to investigate. Multi-modal imaging provides the opportunity to address this challenge.

Radiopeptides have been developed for imaging and therapy and are being used in various cancer types, most notably in neuroendocrine tumors and more recently also in prostate and breast cancers [5–15]. Through coupling of radionuclides to tumor-targeting peptides, the possibility arose to image these tumors using SPECT [11, 14, 16] or PET [17] and treat the tumors by internal irradiation using peptide receptor radionuclide therapy (PRRT). In somatostatin receptor-overexpressing neuroendocrine tumors, radiolabeled somatostatin analogs have shown to be highly effective both in localizing primary and metastatic tumors and in staging and treating these tumors [18, 19]. This approach is mimicked in prostate cancer and breast cancer using bombesin analogs targeting the gastrin-releasing peptide receptors (GRPr) with high affinity [11, 15, 16, 20, 21]. Improvements in therapeutic efficacy are an important goal, which may be assisted by additional information on the intra-tumoral distribution of radiopeptides. In vivo molecular imaging constitutes an important basis for research on tumor-targeting peptides and allows for in-depth analysis of factors that play a crucial role in successful tumor targeting. Previous preclinical imaging studies raised questions concerning in vivo uptake patterns. Despite high receptor expression and homogenous receptor density in our model, assessed by biodistribution and in vitro autoradiography [21], high-resolution in vivo SPECT imaging revealed non-uniform uptake patterns of radioactivity within tumor tissue [20, 22, 23]. Distribution and accumulation of radiopeptides relies on several functional characteristics of both tissue and peptide, such as tissue vascularization and peptide ability to extravasate from blood vessels, high binding affinity for the receptor, and good retention in the tumor. The peptide analog applied in this study has earlier been shown to have high specific affinity for the GRP receptor (Additional file 1: Figure S1), so other factors contributed to the limited and non-uniform tumor uptake seen in vivo [11, 15]. To further identify tissue characteristics and tissue composition of solid tumors that determine peptide distribution, MRI was used as it allows for excellent soft tissue characterization. Furthermore, weighted MR images can be tailored to be sensitive to tissue characteristics by choosing the appropriate imaging parameters, such as edema (T2 scan), necrosis (T2 and T2* scans), and hemorrhages (T1, T2, and T2* scans). Such tissue characteristics all lead to lower tissue density or reduced functionality, which could potentially reveal tissue properties responsible for heterogeneous in vivo peptide uptake. Moreover, MRI also allows investigation of functional tumor characteristics by means of perfusion/permeability measurements using dynamic contrast-enhanced (DCE)-MRI [24–27]. Since vascular properties of the tumor play a role in delivery of radiopeptides to tumor tissue, non-uniform peptide distribution patterns might be related to vasculature function within the tumor. In a previous study, we found a substantial correlation between radiolabeled somatostatin analog uptake in tumors, as determined by SPECT and DCE-MRI-derived tumor perfusion/permeability parameters in a preclinical rat pancreatic-tumor model expressing somatostatin receptors [22]. Tumor areas with low DCE-MRI parameter values corresponded with low peptide uptake.

In the current study, we used solid tumors from a patient-derived xenograft (PDX) of prostate cancer with high expression of GRPr as a model system that appears homogenous on histological H&E stains with high receptor density throughout the tumor as detected by in vitro autoradiography, which led us to expect a high and homogeneous in vivo uptake throughout the tumor. Pilot imaging experiments, however, showed this was not the case. Uptake of radiopeptide highly specific for the GRP receptor, as detected by SPECT, was compared to weighted MR images with various scanner settings and DCE-MRI perfusion measurements to define vascular density and functionality of the tissue.

## Methods

### PC295 animal model

Male NMRI athymic nude mice (*n* = 6, Taconic M&B, RY, Denmark), weighing approximately 30–35 g, were used. PC295 tumor fragments were implanted subcutaneously in the right shoulder. To improve tumor take and growth, mice were given subcutaneous testosterone implants. The average largest diameter of the tumors was 14.1 ± 4 mm at the time of imaging. During imaging, the animals were anesthetized using isoflurane (Nicholas Piramal Limited, London, UK). Body temperature was controlled using a heated scan bed. To ensure the same position of the animal during both SPECT/CT and MRI imaging, animals were placed in an animal holder compatible with both imaging modalities. After completion of the examinations, animals were sacrificed and tumors were resected and frozen for autoradiography and H&E staining. All animal experiments described in this study were approved by the Animal Experiments Committee under the Dutch Experiments on Animal Act and adhered to the European Convention for Protection of Vertebrate Animals used for Experimental Purposes (Directive 86/609/EEC).

### Radiolabeling

Radiolabeling of (DOTA-(βAla)2-JMV594) with indium-111 was performed as described previously [11, 28]. In short, ^111^InCl_3_ (Covidien, Petten, the Netherlands) was added to a mixture of DOTA-JMV, quenchers, and sodium acetate (final pH 4.0–4.5). Quenchers applied were ascorbic acid, (Sigma Aldrich, Zwijndrecht, the Netherlands), gentisic acid (Covidien, Petten, the Netherlands), and methionine (Sigma Aldrich, Zwijndrecht, the Netherlands). The mixture was incubated for 20 min at 80 °C and cooled to room temperature for 5 min prior to quality control. Quality control was performed using HPLC as described [28]. Specific radioactivity used in this study was 100 MBq/nmol. Labeling efficiency was measured through ITLC and was >95 % in all cases.

### SPECT/CT imaging

Two hours prior to SPECT imaging, animals received ^111^In(DOTA-(βAla)2-JMV594) (25 MBq/25 pmol) in a total volume of 100 μl in the tail vein. SPECT/CT images were acquired using a four-head multipinhole NanoSPECT/CT camera (Bioscan, Mediso Medical Imaging Systems, Hungary). SPECT/CT images were acquired using 36 projections, 120 s/projection, and quality factor 1. SPECT images were reconstructed using the OSEM method, with six iterations and a voxel size of 0.4 × 0.4 × 0.4 mm. CT images were acquired using the following settings: 360 projections, 45 kVp tube voltage, 1000 ms exposure time, a scan time of 9 min, and a voxel size of 0.2 × 0.2 × 0.2 mm. In a post-processing step, the CT images were resampled to the SPECT resolution.

### MRI imaging

Directly after SPECT/CT imaging, MR images were acquired. Imaging was performed on a 7.0-T dedicated animal scanner (Discovery MR901, Agilent Technologies/GE Healthcare) using a four-channel surface receiver coil (Rapid MR International, OH, USA) and a 150-mm transmit body coil.

Several weighted images were acquired to study tumor tissue composition. Five T1-weighted scans were acquired using a spin echo sequence with varying repetition times (TR) (200, 400, 800, 1200, and 2400 ms), echo time (TE) of 8.0 ms, 90° flip angle, a 5.0-cm field of view, and a voxel size of 0.2 × 0.2 × 0.6 mm in order to calculate T1 value from the saturation recovery curves [29]. The weighted T2 scan was obtained from the highest TR of the T1 map (spin echo sequence, TR/TE = 2400/8 ms). High-resolution T2* images were acquired using a gradient echo sequence, TR 10 ms, TE 4.5 ms, 6° flip angle, field of view (FOV) 4 cm, and a voxel size of 0.098 × 0.098 × 0.30 mm. The T1 map was additionally used to calibrate the DCE data. The T2* images were used for registration purposes.

Subsequently, DCE-MRI images were acquired. Gadobutrol contrast agent (Gadovist, Bayer, Mijdrecht, the Netherlands) was administered intravenously in a lateral tail vein. A single bolus of 10 μl Gadovist was injected and flushed with 150 μl injection fluid. In order to achieve high temporal resolution, DCE-MRI images were acquired using time-resolved imaging of contrast kinetics (TRICKS): 144 images were acquired with a temporal resolution of 4.7 s each. These images were acquired in three sets of 48 images, 3.76 min each, with intervals of 60 and 120 s between the respective acquisitions, and total measurement time was 14 min. Other acquisition parameters for TRICKS included TR 3.4 ms, TE 1.0 ms, 10° flip angle, FOV 5.0 cm, and a voxel size of 0.2 × 0.2 × 0.6 mm.

### Autoradiography and histology

After imaging, tumors were resected for in vitro autoradiography analysis, by flash-freezing the tumors in liquid nitrogen. Tumors were sliced in 10-μm sections (Microm Cryo-Star HM 560 M, Walldorf, Germany), mounted on glass slides (Superfrost plus slides, Menzelgläser, Braunschweig, Germany), and placed on a phosphor screen (Packard Instruments Co., Meriden, CT, USA). The phosphor screens were digitized after 24 h using the OptiQuant 03.00 image processing system (PerkinElmer, Groningen, the Netherlands). For in vitro autoradiography, the tumor slices were stored at −80 ° C for 30 days to allow for radioactive decay to eliminate background signal. Frozen tumor sections were sliced in 5-μm sections, mounted on glass, and incubated for 1 h with ^111^In(DOTA-(βAla)2-JMV594) (100 MBq/nmol) at 37 °C. Excess radiolabel was removed by a washing step, and the slides were placed on a phosphor screen for 1 h. The screens were digitized, and images were used to visually assess receptor distribution. Adjacent slices to the autoradiography sections were stained with hematoxylin and eosin to study the tissue structure.

### Data analysis

#### Registration of MRI with SPECT/CT

SPECT and CT images were aligned automatically by dedicated software in the SPECT/CT scanner. However, mismatches did occur, and additional manual alignment of SPECT and CT images was performed in all cases. Manually, we aligned the centers of the tumor as seen on CT and on SPECT so that all SPECT signals was contained within the tumor. An example of a matched SPECT/CT is depicted in the supplemental material (Additional file 2: Figure S2). The accuracy is limited to the SPECT voxel level.

To enable spatial correlation between SPECT and MRI, the high-resolution T2* images were registered to SPECT/CT images. The tumors were manually delineated on both the T2*and CT scans. Using these contours, tumor masks were generated. The T2* masks were registered to the CT masks using Elastix [30] applying subsequently a rigid and an affine registration scheme. We registered the masks as delineated on the CT and T2* datasets using a rigid and affine registration scheme. We did not perform deformable registrations since the shapes of both masks were very similar and since the information content within the CT masks was very low. The transformation parameters acquired from both registration schemes were then used to transform the DCE-MRI images to the CT images.

### DCE-MRI quantification

For calculation of DCE-MRI-derived parameters, a semi-quantitative as well as a quantitative analysis method was used. The analysis was performed using in-house developed software based on MATLAB (MathWorks, Natick, MA, USA).

#### Semi-quantitative analysis

Signal-enhancement-over-time curves (SI-curves) were constructed for all voxels inside the tumor mask. Six semi-quantitative parameter maps were calculated directly from the SI-curves: (1) maximum enhancement (Smax), (2) time to peak (TTP), (3) area under the whole curve (AUC), (4) area under the curve for the first 60 s (AUC60), (5) wash-in (slope of the curve from the time of injection to the first peak), and (6) wash-out. Since the signal intensity in MRI is relative and can differ between subjects and scanners, semi-quantitative parameters were normalized to pre-contrast levels, enabling comparison between the different animals.

#### Quantitative analysis

To model the pharmacokinetic behavior of contrast agent inside the tumor, the standard compartment model of Tofts et al. [27] was used. The two parameters of interest in this model are *K*^trans^ and *k*_ep_. *K*^trans^ is the capillary transfer constant from the blood pool into tissue and is influenced by blood flow and capillary permeability inside the tumor. Its physiological interpretation therefore depends on the balance between perfusion and permeability [27]. *k*_ep_ is the contrast exchange rate from the extracellular extravascular space to the blood.

In order to calculate quantitative DCE-MRI parameters, absolute MRI values are needed. For this purpose, T1(0) maps were calculated from the five T1-weighted scans for each animal. Based on all maps, a mean T1(0) value was calculated from individual tumors and the averaged T1(0) from the five mice was used to convert the SI-curves to contrast-concentration curves. In addition, the Tofts model requires an arterial input function (AIF), which was taken from literature for nude mice [31]. We injected a slightly higher amount of Gd-DOTA at a similar rate: Weidensteiner: 0.1 mmol/kg at a rate of 0.05 ml/s and we injected 0.3 mmol/kg at a rate of 0.04 ml/s. The compartment model was then fitted to the contrast-concentration curves using the standard Tofts equation, resulting in parametric maps of *K*^trans^ and *k*_ep_.

### Correlating DCE-MRI-derived parameters with SPECT peptide uptake

We investigated the spatial correlation between radioactive peptide uptake as measured with SPECT and DCE-MRI-derived parameters using the Spearman’s rank correlation coefficient. The correlations were calculated per mouse dataset. The correlation was calculated for each tumor individually using all voxels within the tumor. The mean is the average of the six individual correlation outcomes. Only voxels within the tumor boundary were taken into account, excluding the tumor-feeding vessels. However, as shown by others [32], the standard-compartment Tofts model sometimes poorly fits for voxels with low contrast enhancement. In these instances, *k*_ep_ has very high, non-physiological values, which were seen to correspond with little to no contrast accumulation on the DCE-MR images. For these voxels, *K*^trans^ and *k*_ep_ were set to zero.

## Results

The prostate cancer xenograft model PC295 was used as a model system to assess the value of DCE-MRI-derived parameters in studying the heterogeneous uptake of radiolabeled peptide ^111^In(DOTA-(βAla)2-JMV594)in tumor tissue as imaged by SPECT.

**Fig. 1:**
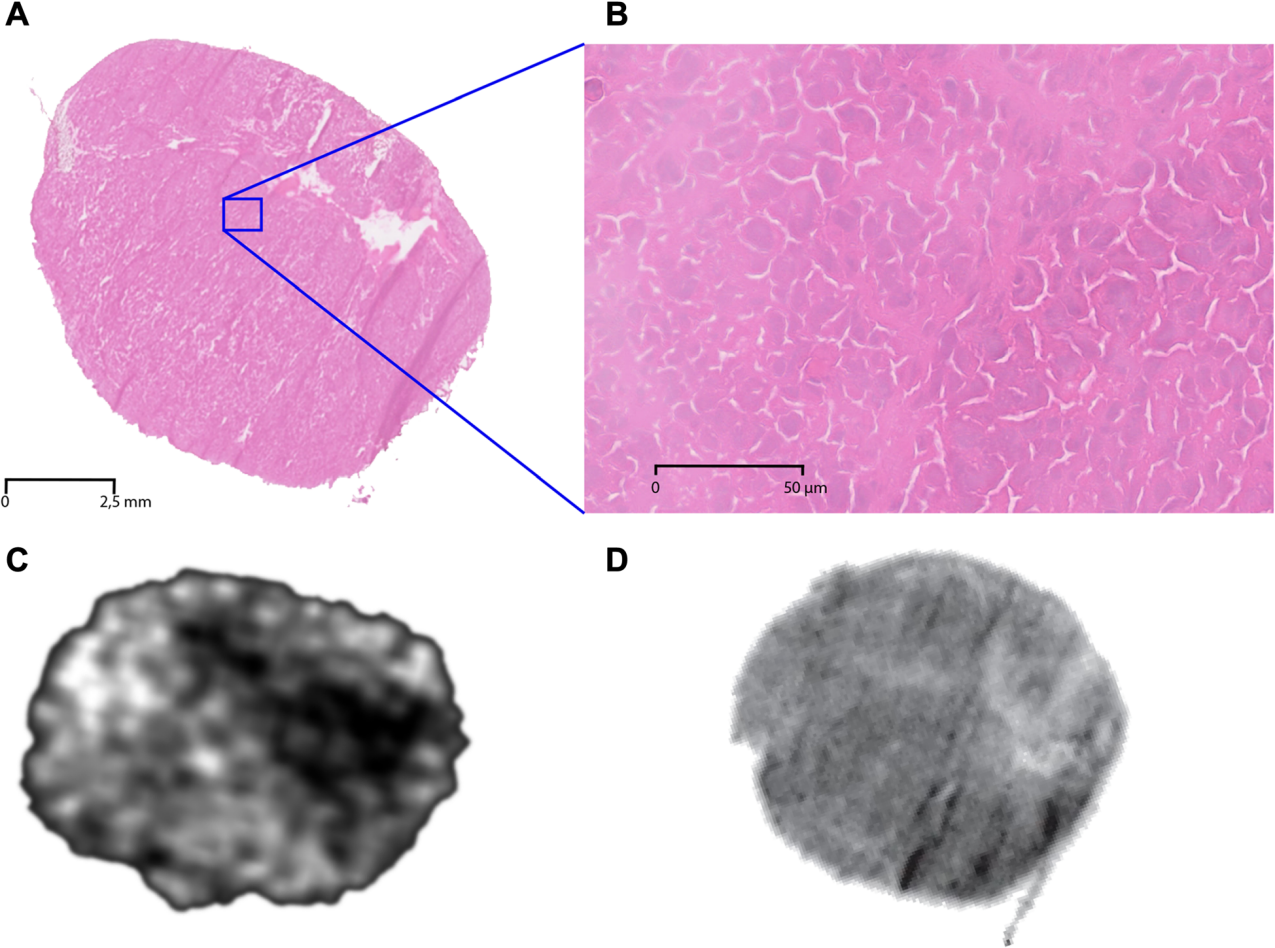
H&E staining of a PC295 tumor showing homogenous tissue density (**a**) and viable tissue as seen on an enlarged section (**b**). Single tumor slice from SPECT image demonstrating heterogeneous peptide distribution in vivo (**c**) despite high and homogeneous receptor density throughout the tumor as assessed by in vitro autoradiography (**d**)

In all PC295 tumors, in vitro autoradiography showed a homogeneous receptor density throughout the tumor and also on histology (H&E staining), homogenously viable tissue was found without necrotic areas (Fig. 1 shows a representative example). In contrast, SPECT images showed a heterogeneous radioactivity distribution. Figure 1 shows slices of approximately the same location. As there is a difference in resolution/slice thickness between the in vivo image and the sections, however, the exact same location cannot be matched. We accomplished this by extruding the tumor and embedding it in the same orientation as the MRI slices (axial) as accurately as possible.

**Fig. 2:**
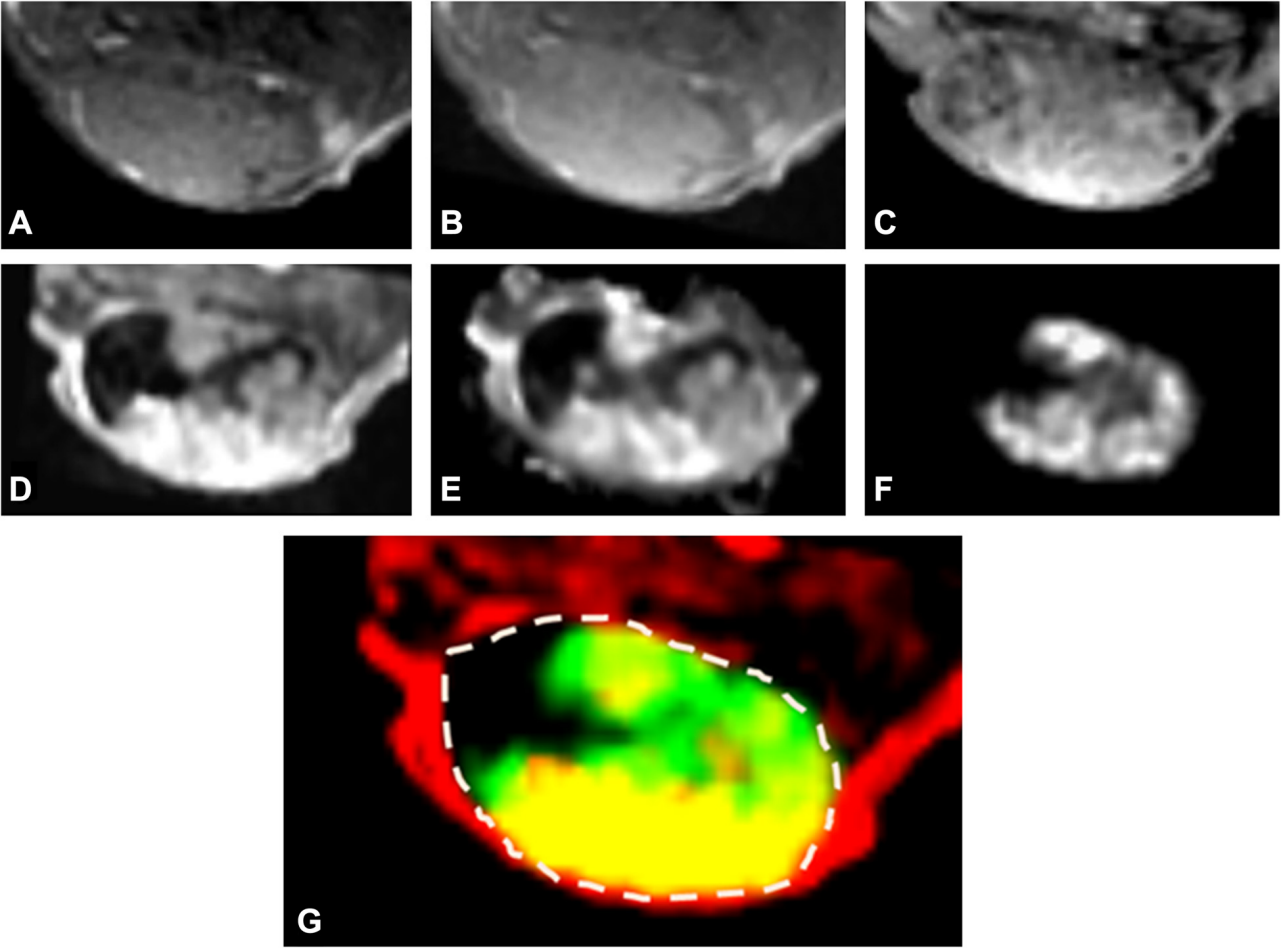
Example of MRI and SPECT images and DCE-MRI parameters of a single tumor slice. **a** T1-weighted MRI. **b** T2-weighted MRI. **c** T2*-weighted MRI. **d** Area under the curve for the first 60 s (AUC60). **e** *K* ^trans^. **f** SPECT radiopeptide uptake. **g** Overlap (*yellow*) between AUC60 (*red*) and SPECT (*green*) within the tumor boundary (*dotted line*)

T1/T2 images of all PC295 tumors revealed a homogeneous tissue composition with no interfering factors caused by edema (T2), necrosis (T2 and T2*), and hemorrhaging (T1, T2, and T2*). All images showed almost uniform signal intensity throughout the tissue as illustrated for one tumor slice in Fig. 2a–c. This is in concordance with the H&E results, where the cell-nuclei staining shows the tissue properties in high resolution, unachievable by MRI, whereas MRI was used for full-coverage analysis of the tumor.

In contrast to the weighted images, DCE-MRI showed a heterogeneous accumulation of contrast agent in most tumors for the DCE-MRI-derived parameters AUC60 and *K*^trans^ (Fig. 2d–f). Overlap (yellow) between SPECT peptide uptake signal (green) and DCE-MRI AUC60 data (red) is shown in Fig. 2g. In general, tumor areas with low DCE-MRI values corresponded with low radioactivity, while high DCE-MRI values corresponded with high radioactivity.

To quantify the spatial correlation between radioactivity and DCE-MRI parameters, the Spearman’s rank correlation coefficient *ρ* (Table 1) was used, which calculates how two variables relate to each other and the strength of the relationship. The spearman coefficients are all moderate to high showing a positive relationship between perfusion of the tissue and the amount of radioactivity, with some parameters showing a stronger correlation between vascular functionality and radiopeptide targeting than others. The DCE-MRI parameters AUC and Smax, representative for contrast agent build-up, were shown to yield a lower correlation with SPECT data than AUC60, wash-in, *K*^trans^, and *k*_ep_, representing the exchange of contrast agent between tissue and blood. The wash-out and TTP parameters showed lower correlations as compared to the other “exchange-related” parameters.

**Table 1:** Means and standard deviations (SD) of Spearman’s correlation coefficient (*ρ*) between SPECT radioactivity data and various DCE-MRI parameters, calculated for 6 mice

DCE-MRI parameter	Mean *ρ* (range)	SD
AUC	0.29 (−0.21–0.52)	0.26
AUC60	0.57 (0.44–0.80)	0.12
Smax	0.40 (0.15–0.64)	0.16
TTP	0.43 (0.0–0.77)	0.26
Wash-in	0.55 (0.41–0.79)	0.14
Wash-out	0.33 (0.08–0.61)	0.22
*K* ^trans^	0.60 (0.39–0.80)	0.14
*k*_ep_	0.63 (0.47–0.79)	0.11

## Discussion

In this study, we investigated several morphological and functional characteristics of tumor tissue that are thought to be related to uptake and distribution of tumor-targeting radiopeptides in a solid-tumor model. Relevant tumor features, such as tissue morphology, vascularization, and functionality of tumor vasculature, were studied using weighted MRI and DCE-MRI. The PC295 tumor used in this study showed promising morphological characteristics, with high tissue viability and high and homogenous GRPr expression (Fig. 1d and Additional file 1: Figure S1), leading to an expected high accumulation of the targeting radiopeptide. In vivo high-resolution SPECT findings revealed, however, that peptide binding was not consistent in all areas of the tumor (Fig. 1, Additional file 2: Figure S2). In vivo tissue characterization with MRI showed that whereas the weighted images showed a homogeneous tissue composition, parameters derived from DCE-MRI correlated well with SPECT values, indicating the relevance of functionality of the tumor vasculature in peptide distribution.

Using a small molecular weight contrast agent, DCE-MRI allows for visualization and quantification of the perfusion/vessel permeability of the tumor vessels. In contrast to weighted MR images, DCE-MRI revealed heterogeneous vascular functionality in all tumor cases. Through voxel-based registration of SPECT values and DCE-MRI-derived parameters, a clear correlation between localized tissue perfusion parameters and radioactivity accumulation in the tumor could be established (Fig. 2). This was most apparent in the exchange-related parameters AUC60, wash-in, *K*^trans^, and *k*_ep_ for all datasets.

To the best of our knowledge, this is the first report on the correlation between radiopeptide accumulation in a patient-derived xenograft and DCE-MRI parameters. This study used GRPr-targeted radiopeptides and a GRPr-positive xenograft model as model systems, but we assume that the results also apply to other (radio)peptides that target receptors highly expressed on solid tumors. Our current findings are comparable with the results obtained in a syngeneic rat neuroendocrine tumor model (CA290948) expressing high numbers of somatostatin receptors but with extensive hemorrhaging and necrosis as observed on histology [22]. In contrast, the current prostate cancer xenograft model consisted of rather solid, slow growing, well-differentiated glandular tissue, which may better reflect solid tumor features in patients.

To study peptide distribution and accumulation in solid tumors, we choose to study GRPr-targeting radiopeptides that bind with high affinity to GRPr-expressing tumor cells It has been shown in several prostate cancers and in PDX models of prostate cancer that the GRPr expression is high [9, 11, 13, 20, 21]. The GRPr antagonist (^111^In(DOTA-(βAla)2-JMV594)) was selected as peptide as it has high receptor affinity and showed prolonged attachment to the cell-surface receptor upon binding [11].

Adequate delivery of peptide to the tumor is an important factor for successful tumor target. Such delivery of peptides is dictated by a combination of the presence of sufficient vasculature as well as of its functional properties, i.e., allowing extravasation from the blood pool to the extravascular extracellular space. Extravasation is the resultant of vascular perfusion and vessel permeability characteristics and can be measured using DCE-MRI. Histological staining of vessels, such as with CD31, could further show the presence of vessels within the tumors and could serve as validation of the relationship between vascularity and radiopeptide accumulation in tumors. Histology, however, cannot display the rate at which perfusion takes place, which the in vivo functional measurements do. Additionally, intra-tumoral pressure also plays a role in the exchange across the vessels and also contributes to the measured DCE-MRI parameters. These functional perfusion/permeability properties of the vascular bed in the tumor tissue can be measured over time using a small molecular contrast agent. In a solid and histologically homogeneous tumor, tissue perfusion is expected to be homogenous as well, as uniform tissue appearance suggests homogenous delivery of nutrients and oxygen. Clearly, functional imaging of the tumor vasculature revealed that this is a simplified perception and that contrast agent build-up in these tumors was not uniform in all areas of the tumor. This lack of uniform vasculature function, and consequently of radiopeptide distribution, was most apparent in the exchange-related parameters (AUC60, wash-in, *K*^trans^, and *k*_ep_) for all datasets. The wash-out and TTP parameters showed lower correlations as compared to the other exchange-related parameters, such as AUC60, *K*^trans^, and *k*_ep_. We showed that the build-up of contrast agent in the PC295 tumors was very slow, and in many areas, little wash-out was visible at the end of the scanning time, resulting in a poor correlation of wash-out and TTP with SPECT uptake.

There are a number of factors that are challenging in this multi-modal imaging approach. Registration between SPECT/CT and MRI is not a simple step, due to small, but significant changes in animal position, even though the animal was kept in the same position for the different modalities using the same holder. SPECT and CT images were aligned automatically by dedicated software in the SPECT/CT scanner. The CT scan, which was acquired along with the SPECT image, was used to register SPECT/CT to MRI. However, visual inspection of the registration between SPECT and CT revealed that this automatic registration did not accurately register all images; therefore, accurate manual registration was required in all cases.

There are several intrinsic factors that might affect the correlation between SPECT and DCE-MRI-derived data, which need to be taken into account. The pharmacodynamic processes imaged by DCE-MRI and SPECT are different. The DCE-derived parameters are a measure of the free exchange of contrast agent between the blood pool and extravascular extracellular space, whereas with SPECT, the accumulation of cell-surface receptor-bound peptides is measured, a dynamic process that is not only affected by peptide delivery via the vasculature but also additionally by peptide receptor-binding affinity of the cell-surface receptors. In addition, DCE-MRI and SPECT measurements are acquired at different time points after administration. SPECT images are acquired 2 h after injection, whereas the DCE images are acquired during the first 10 min of contrast injection. MRI-derived parameters and SPECT uptake values are therefore not measuring the same processes. Moreover, this SPECT peptide is larger than the MRI contrast agent, but both are considered small molecules (molecular weight ^111^In(DOTA-(βAla)2-JMV594) conjugate—1641.90 [11], molecular weight Gadovist—604.7), and at this size considering the vessel characteristics in tumors, the leakage characteristics are not expected to differ greatly.

Nevertheless, these data showed that DCE-MRI using contrast agent to measure perfusion appeared to be a good indicator for the ability of the radiopeptide to leak into the targeted tissue. In areas with low DCE-MRI parameters, indicating low perfusion, the radiopeptide seemed to have poor ability to reach that region and, consequently, showed poor uptake. Higher DCE-MRI parameter values correlated with higher radiopeptide accumulation indicating that good tissue perfusion and blood vessel permeability is essential for peptide delivery into tumor tissue.

The high correlation coefficients of the peptide accumulation (SPECT) and the DCE-MRI parameters AUC60, wash-in, *K*^trans^, and *k*_ep_ further underscored the importance of vascular extravasation and the relationship of local tumor-vessel functionality with peptide delivery to the cells. This result reveals the importance of establishing perfusion and permeability properties of solid tumors prior to radiopeptide-targeted interventions. In a patient study, the tumor-vascular functionality measured by DCE-MRI showed predictive value for PRRT treatment efficacy, in which patients with large tumor areas of low perfusion responded worse to therapy [33]. Improvements of tissue perfusion or optimal timing of treatment at time points with the highest DCE-MRI parameters could help to increase overall tumor dose and consequently improved PRRT efficacy. Better targeting efficiency could also allow lower administered doses, which in turn can benefit patients concerning healthy organ toxicity [34–36].

Preclinical in vivo studies investigating tumor targeting by high-affinity peptides are predominantly performed in rats and mice using biodistribution data to quantify the amount of radioactivity in the tumor. Yet, these studies do not take into account the influence of tissue perfusion of the respective peptide, which could lead to misinterpretation of radiopeptide-targeting efficacy. Acquiring DCE-MRI parameters for these tumors may help to better interpret such radiopeptide uptake and biodistribution results.

## Conclusions

In conclusion, we have shown that peptide delivery and accumulation in solid tumors as visualized by SPECT is correlated to vascular extravasation and tumor-vessel functionality as assessed by DCE-MRI parameters.

Despite differences in pharmacokinetic behavior of MRI contrast agent and SPECT peptide, this study showed that the perfusion/permeability characteristics as determined with DCE-MRI can provide predictive information on the ability of radiopeptides in reaching the targeted tissue [22]. The combined use of SPECT and MRI can therefore add crucial information on the local perfusion efficiency in tumors. We believe perfusion to be an essential and as yet an underestimated factor in tumor uptake in peptide-targeted imaging and therapy. It remains to be further investigated if DCE-MRI-derived parameters may relate to PRRT efficacy.

## Ethics approval

All applicable international, national, and/or institutional guidelines for the care and use of animals were followed.

## Additional files

Additional file 1: Figure S1. Autoradiography of PC295 xenograft incubated with 10^-9^ M [^111^In]JMV4168. Left: homogenous GRPr expression in PC295. Right: blocked section, showing specific uptake throughout the whole tumor. ^115^In-labeled JMV was prepared by addition of a >5 times mol ratio of ^115^In to the concentration of peptide in the reaction mixture. After labeling, labeled peptides were injected into a HPLC. Chromatogram at 278 nm showed base-to-base separation between labeled and non-labeled DOTA-JMV4168. Duplicate cryostat sections (10 μm) of PC-295 xenograft where incubated for 1 h with 10^-9^ M [^111^In]JMV4168, for blocked section with addition of 1000× excess of cold labeled peptide. The receptors where visualized by autoradiography using phosphor imaging screens and the Cyclone phosphor imager, and data were analyzed using OptiQuant software (Packard Instruments Co., Groningen, the Netherlands). [^111^In]JMV4168 uptake could be blocked almost completely with an excess of cold labeled peptide, illustrating the specific binding of this compound. (TIF 276 kb) Additional file 2: Figure S2. Cross section of a mouse bearing a subcutaneous tumor. As can be seen, the tumor to background ratio is high, and after 2 h, the blood pool is under the detection limit, visible in the cross section of the heart. The figure additionally shows the high in vivo resolution that allowed the analysis of all the areas with different amounts of radiopeptide uptake levels. (TIF 165 kb)
