# Supplementary figures and images for: Imaging heterogeneity of peptide delivery and binding in solid tumors using SPECT imaging and MRI
Source: EJNMMI Res. 2016 Jan 14;6:3. doi: 10.1186/s13550-016-0160-4 (PMC4713394; doi:10.1186/s13550-016-0160-4)

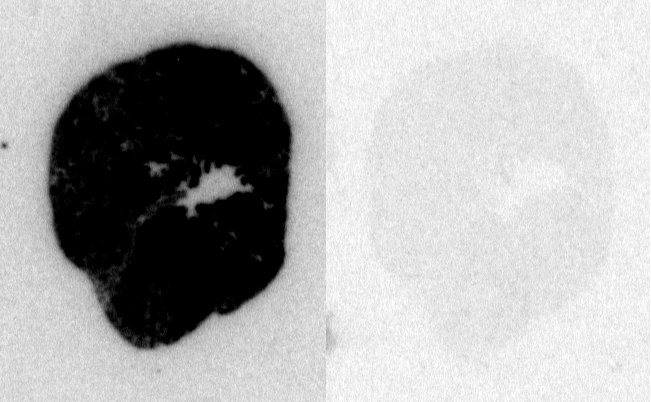

Supplement: Additional file 1: Figure S1. — Autoradiography of PC295 xenograft incubated with 10-9 M [111In]JMV4168. Left: homogenous GRPr expression in PC295. Right: blocked section, showing specific uptake throughout the whole tumor. 115In-labeled JMV was prepared by addition of a >5 times mol ratio of 115In to the concentration of peptide in the reaction mixture. After labeling, labeled peptides were injected into a HPLC. Chromatogram at 278 nm showed base-to-base separation between labeled and non-labeled DOTA-JMV4168. Duplicate cryostat sections (10 μm) of PC-295 xenograft where incubated for 1 h with 10-9 M [111In]JMV4168, for blocked section with addition of 1000× excess of cold labeled peptide. The receptors where visualized by autoradiography using phosphor imaging screens and the Cyclone phosphor imager, and data were analyzed using OptiQuant software (Packard Instruments Co., Groningen, the Netherlands). [111In]JMV4168 uptake could be blocked almost completely with an excess of cold labeled peptide, illustrating the specific binding of this compound. (TIF 276 kb) [file 13550_2016_160_MOESM1_ESM.tif]

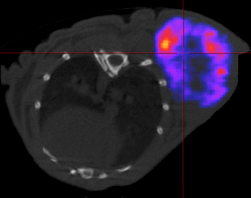

Supplement: Additional file 2: Figure S2. — Cross section of a mouse bearing a subcutaneous tumor. As can be seen, the tumor to background ratio is high, and after 2 h, the blood pool is under the detection limit, visible in the cross section of the heart. The figure additionally shows the high in vivo resolution that allowed the analysis of all the areas with different amounts of radiopeptide uptake levels. (TIF 165 kb) [file 13550_2016_160_MOESM2_ESM.tif]
